# Seeking systems-based facilitators of safety and healthcare resilience: a thematic review of incident reports

**DOI:** 10.1093/intqhc/mzae057

**Published:** 2024-06-25

**Authors:** Catherine Leon, Helen Hogan, Yogini H Jani

**Affiliations:** Department of Health Services Research and Policy, London School of Hygiene & Tropical Medicine, London WC1H 9SH, United Kingdom; Department of Health Services Research and Policy, London School of Hygiene & Tropical Medicine, London WC1H 9SH, United Kingdom; Department of Practice and Policy, University College London School of Pharmacy, London WC1N 1AX, United Kingdom; Centre for Medicines Optimisation Research and Education, University College London Hospitals NHS Foundation Trust, London, NW1 2BU, United Kingdom

**Keywords:** patient safety, medication safety, risk management, governance, incident reporting

## Abstract

Patient safety incident reports are a key source of safety intelligence. This study aimed to explore whether information contained in such reports can elicit facilitators of safety, including responding, anticipating, monitoring, learning, and other mechanisms by which safety is maintained. The review further explored whether, if found, this information could be used to inform safety interventions. Anonymized incident reports submitted between August and October 2020 were obtained from two large teaching hospitals. The Systems Engineering Initiative for Patient Safety (SEIPS) tool and the resilience potentials (responding, anticipating, monitoring, and learning) frameworks guided thematic analysis. SEIPS was used to explore the components of people, tools, tasks, and environments, as well as the interactions between them, which contribute to safety. The resilience potentials provided insight into healthcare resilience at individual, team, and organizational levels. Sixty incident reports were analysed. These included descriptions of all the SEIPS framework components. People used tools such as electronic prescribing systems to perform tasks within different healthcare environments that facilitated safety. All four resilient capacities were identified, with mostly individuals and teams responding to events; however, monitoring, anticipation, and learning were described for individuals, teams, and organizations. Incident reports contain information about safety practices, much of which is not identified by traditional approaches such as root cause analysis. This information can be used to enhance safety enablers and encourage greater proactive anticipation and system-level learning.

## Introduction

Incident reporting and learning systems in healthcare serve as repositories of voluntary reports of events that lead to actual or potential harm to patients, and they are a key source of safety intelligence [1–4]. Learning from these reports may be derived through national, organizational, or local analysis, e.g. by a ward or teams within a hospital [2]. Traditionally, incidents are scrutinized to identify contributory factors and develop interventions to prevent recurrence [4, 5]. However, this approach has been challenged for focusing on unintended outcomes, thus limiting learning opportunities [6, 7]. By seeking to understand what went wrong, learning from incidents can narrow its focus on problems that need to be fixed, often relying on applying remedial strategies to the individuals involved [5, 8].

Healthcare is complex and unpredictable. Safety is maintained by individuals, teams, and organizations adapting to differing circumstances, a concept known as healthcare resilience [9–12]. Healthcare takes place within a work system comprising of interacting components including: (among others) patients, caregivers, and staff, the tasks required to deliver care, available tools and technology, and aspects of the care environments [11]. Safety improvements can be developed by exploring the work system and the resilient adaptations taking place within it [6, 10, 13, 14]. Resilient activities include monitoring for issues that may impact safety, responding to situational changes, anticipating potential issues, and learning from new information [15]. Work system complexities and resilient adaptations contribute to all outcomes experienced in healthcare; therefore, learning should be sought from where care was successful, where harm was prevented (near-misses), and where harm occurred [9, 14, 16]. This understanding of safety has been termed Safety-II [17].

Less attention has been given to whether incident and associated investigation reports can provide information on healthcare resilience and other safety facilitators. Although reports focus on adverse events, they may also describe factors that support safety or resilient activities undertaken. If identifiable within incident reports, these data could expand the use of incident analysis beyond learning from factors contributing to harm towards learning from successful aspects of the system.

This research aimed to determine whether incident analysis can identify aspects of the work system and resilient activities that facilitate safety. Anticoagulants, known to be high-risk medications for patient safety, were used to trial the approach.

The objectives were to explore whether incident reports can be used to identify:

- Work system factors facilitating anticoagulant safety.
- Resilience activities (known as potentials) at individual, team, and organizational levels.
- Practical insight into areas for safety improvement.

## Methods

A qualitative, thematic review of the narrative content of anticoagulant-related incident reports was undertaken.

The study was conducted in England, using incident report data from two large teaching hospitals reported between August and October 2020. Although reporting systems differed between organizations, both contained the core set of information required by all reporting systems in England [18]. Those used for the analysis included the location of the incident, the type of incident (e.g. medication, staff, or property), the level of harm experienced by the patient, narrative descriptions of the event, and the initial actions taken. Many incident reports included the investigation by the local manager.

### Ethics and other permissions

Ethics approval was granted by the London School of Hygiene & Tropical Medicine Ethics Committee (Ref. 22980). NHS ethics approval was not required because the data were anonymized. The project was registered at both hospitals as a service improvement project and followed local data governance requirements.

### Sampling strategy

A structured search using medication names, commonly used abbreviations, and keywords was performed in each incident reporting system to identify all incidents relating to anticoagulation between August and October 2020. The full list of search terms is included in the supplementary material.

Extracted incidents were manually checked for anonymity and redacted to remove any reference to patients, staff members, hospital, or location. They were numbered chronologically. The two hospitals used different low-molecular weight heparin (LMWH) products, dalteparin and enoxaparin; therefore, these were anonymized by describing them only as LMWH, with associated doses in each incident changed to Dose A, Dose B, etc.

### Inclusion and exclusion criteria

To be included, reports had to relate to patient care and any anticoagulant medication. Incidents were excluded if they did not contain sufficient narrative information to understand the events. All fields in the extracted data, including the local investigation, where available, were included to aid in understanding the narrative and context of the incident.

### Data synthesis

**Figure 1:**
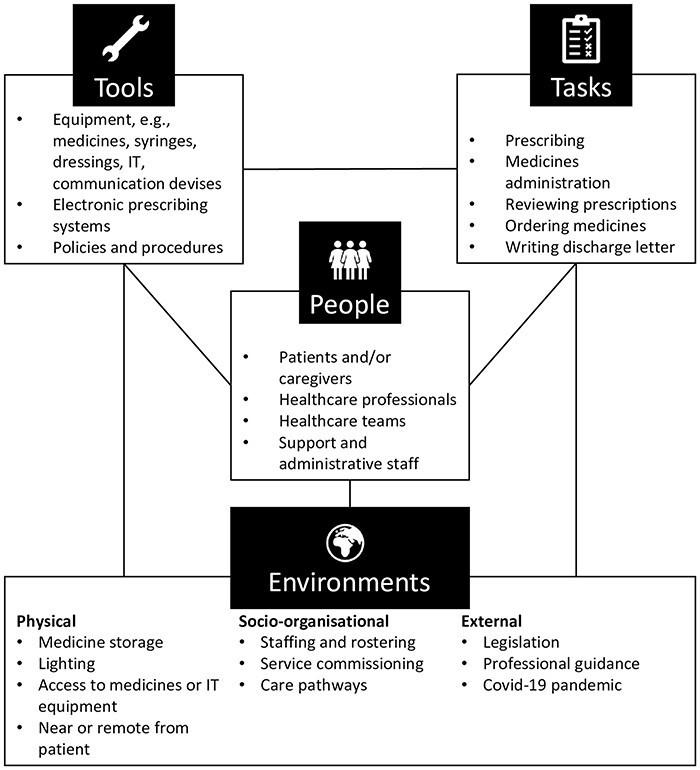
The SEIPS 101 work system with examples of each factor [21]

A framework approach to coding was used, guided by two frameworks: the Systems Engineering Initiative for Patient Safety (SEIPS) [11, 19–21] and the four resilience potentials [15]. SEIPS was used to categorize different work system components described in the narrative according to people, environments, tools, and tasks (Fig. 1). The second framework was used to identify resilience potentials: respond, monitor, anticipate, and learn. Responding was defined as actions taken in response to an event. Monitoring was defined as the identification of actual events through the knowledge and experience of staff. Anticipation was the identification of potential or future issues by staff, and learning incorporated any reflections on actions taken in response to an event that might prevent similar issues in the future.

Incidents were analysed line by line by one author to identify SEIPS components involved and evidence of anticipation, monitoring, responding, or learning. Where work system factors were identified, those that facilitated safety were listed in a separate column in a spreadsheet. Resilience potentials were recorded in the same way and were further classified according to whether these were at individual, team, or organizational levels. This insight into the context, terminology, processes, and systems within the respective organizations improved the interpretation of what might have otherwise been ambiguous narratives. The coding and themes were discussed with co-authors to enhance data validation. In cases of disagreement, the mapping was reviewed, and consensus was reached.

## Results

**Figure 2:**
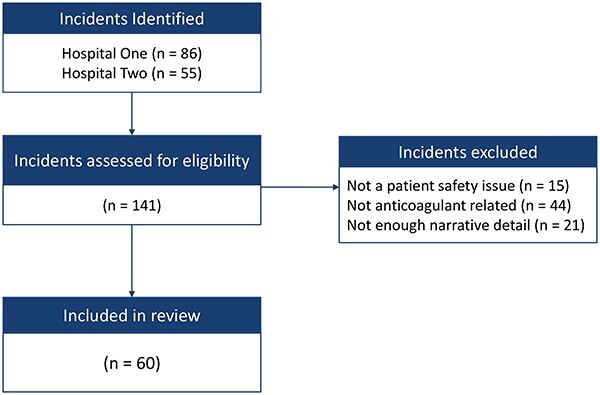
Incident selection following the application of inclusion and exclusion criteria

Overall, 141 incidents were identified, 86 from Hospital A and 55 from Hospital B. After applying the inclusion and exclusion criteria (Figure 2), 60 incidents were included in the analysis. Many incidents were excluded as these were not anticoagulant related, but the ward in which they occurred shared an abbreviation with a search term.

Two-thirds (*n* = 41) of the incidents involved LMWH, nine warfarin, four apixaban, two edoxaban, and one each involved heparin and rivaroxaban. Incidents occurred in a variety of clinical areas including emergency units, medical and surgical wards, specialist areas, and one in a patient’s home. Patients experienced no harm according to 52 reports, with four near-misses, minor harm twice, and moderate harm twice.

**Figure 3:**
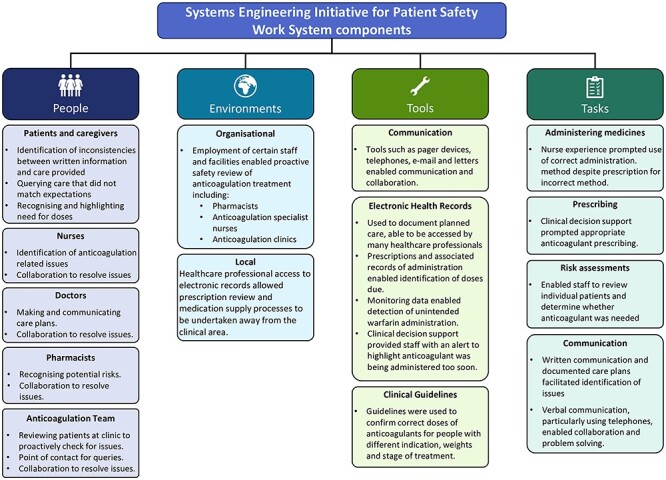
Work system components and their role in facilitating safety

The SEIPS work system factors that were identified and how they facilitated safety are described below. A summary is available in Fig. 3. Although reported separately, the combination of factors and interactions between all the people involved, using the tools available to perform tasks within their healthcare environments, impacted safety.

### Facilitators of safety

#### People

People, including patients and staff, facilitated safety using their knowledge, experience, and skills to identify problems. They demonstrated tenacity, communication, and collaboration to resolve issues or adjust plans in response to problems.

For example, staff adapted when they were unable to administer anticoagulants to a patient in her home as intended.

Patient attended ED and was diagnosed with both Covid pneumonia … and a new [pulmonary embolism (PE)]. The plan was for the patient to go home and self-isolate, and for ambulatory care to counsel patient and initiate anticoagulation therapy for the PE; ambulatory care cannot do this whilst the patient is housebound with Covid. She was discharged with a single dose of [LMWH] …. We have had to organise a courier to deliver [LMWH] to her home to continue her treatment until the quarantine is over.

#### Environments

Organizational resources supporting safety were identified. Follow-up processes, such as clinic appointments or healthcare professional reviews, allowed monitoring of at-risk patients to prevent problems. The pharmacist’s clinical review of the medication chart allowed confirmation that medications were prescribed safely.

Communication channels enabled the escalation of potential issues and prevented harm.

Telephone call from out-of-area GP to anticoagulation service … regarding patient discharged on warfarin and lack of handover and discharge information.

Organizational enablers supported such communication, e.g. by providing contact details for the specialist anticoagulation team.

#### Tools

Clinical guidelines and electronic health records (EHRs) were the most frequently described tools enabling anticoagulation safety. Guidelines were used to influence individualized prescriptions and aid their review to prevent or identify potentially life-threatening incorrect dosing.

EHRs facilitated safety both in terms of the information they contained (patients medical notes, prescriptions, and administration records) and the availability of clinical decision support. EHR rules and alerts supported guideline-based prescribing and highlighted potential errors to users, often in real-time. In one report, a pharmacist identified a patient with an incorrectly documented weight based on an estimate and the higher than indicated LMWH dose prescribed consequently. EHR clinical decision alerts highlighted issues, enabling staff to act swiftly to prevent or mitigate adverse events.

Patient … had been prescribed … [LMWH] prophylaxis. After giving the medications and signing them it popped up with a message to say [LMWH] had already been given at 0852 ….

#### Tasks

Communication was the most frequently described task promoting safety. Examples included querying prescriptions, clarifying, and collaborating to update plans following unintended events. This example captures the complexity of such communications across secondary, primary, and home care settings.

Patient attended outpatients’ area to be seen in our clinic however we were not on site. Full bloods taken - noted INR to be high as patient no longer on warfarin. Contacted patient’s GP for more details. Medications managed by care assistants according to GP.

Contacted patient’s care assistant - she states that the patient has several boxes of different medications at home including [warfarin] which he had been taking since discharge alongside prescribed [direct oral anticoagulant].

The clinic attendance provided the opportunity to review this man’s care. Remote access to EHR notes and blood tests enabled the anticoagulation team to identify the unintended continuation of warfarin. Subsequent communication took place the General Practicitioner, community pharmacist, anticoagulation team, and care assistants to develop a plan to support the patient in taking his medications safely.

### Resilient activities identified

#### Respond

Incident reports, by their nature, describe an individual’s actions to resolve or manage situations that are encountered. Therefore, many demonstrate responding, particularly at an individual level. Here, a member of the anticoagulation team provided advice to avoid harm.

Patient was given [higher dose A LMWH] instead of [lower dose B]. Informed the nurse in charge, on call doctor and on call pharmacy. Recheck patient vital signs and monitor patient for any bleeding.

#### Learn

Learning was identified at individual, team, and organizational levels.

Reflected on my own practice - when I had a phone call with the patient I asked if he was on any ‘blood thinning’ medication and perhaps I didn’t make this clear what I meant. Also was during a phone call when I had given the patient a lot of information about stem cell transplant plans so he may have felt overwhelmed etc. Spoke to [patient’s] usual haematology team at [another hospital] and we have discussed how we can improve communication going forward.

Team learning was observed in reports that described reviews of system processes such as EHR use.

This was an unusual scenario .… The patient had such an effective diuresis that he lost over 30kg, which placed him in a different dosing category for [LMWH]. The team as a whole have reflected upon this and whether using the ‘ward round’ feature of EPR might mitigate in the future.

Organizational learning was demonstrated where whole pathways or clinical guidelines were updated in response to issues raised, e.g. changes to ambulatory care provision to ensure that treatment was available to those self-isolating due to COVID-19.

#### Anticipate

Team-level anticipation was demonstrated when automatic email replies were used to highlight the correct referral method.

Patient seen … with suspected [deep vein thrombosis (DVT)]. He was referred to the DVT clinic for investigation, however the doctor emailed the anticoagulation clerical team email address which (as per the out-of-office reply the doctor would have received) is not checked out of hours.

At an organizational level, evidence of anticipation was seen by clinical decision support alerts designed to highlight the known risk of duplicate dosing.

#### Monitor

Individuals used their knowledge, experience, and/or skills to monitor for issues. For example, a pharmacist identified that an anticoagulant medication was not kept in the clinical area and would need to be supplied by the pharmacy. A patient contributed to his safety by letting the staff know that he had missed a dose of his usual anticoagulants before admission.

Patient came…to have some bloods taken …. Whilst here he asked me to give his Filgrastin injection and a dose of Tinzaparin as his district nurse had not been able to get to him before he left for the hospital.

## Discussion

### Statement of principal findings

Incident reports can provide insight into interacting factors that promote safety and aspects of healthcare resilience that can be used for safety improvement. People identified and responded to safety issues through communication and collaboration. Organizational resources, such as staff with responsibility for reviewing anticoagulants (anticoagulation teams and pharmacists), supported safe care. Clinical guidelines, EHRs, and communication devices were tools for enhancing anticoagulation safety. The initial investigations undertaken by the local manager provided additional descriptions of many system-based factors. Incident reports are subjective accounts, written after events, and therefore the most resilient activities identified were of individuals responding to unanticipated events; however, aspects of monitoring, anticipation, and learning were also detected.

Our analysis identified system factors frequently associated with safer care. For example, communication channels often enabled issues to be resolved. Opportunities therefore arise to improve safety at a system level by supporting these key areas, an example of proactive learning from successful outcomes. The approach also highlights where staff are regularly adapting to situations because of suboptimal factors in the work system. These adaptations provide insight into where safety risks exist and where potential interventions to prevent harm might be needed. Multiple incident reports described staff recognizing incorrect anticoagulation doses for specific patients. Repeated resilient adaptations for this recurring risk highlighted the need to evaluate how other safeguards, such as clinical decision support tools and guidelines, could enable systems to anticipate this issue and prevent it in the future. In England, the introduction of the Patient Safety Incident Response Framework [22] and the Learning from Patient Safety Events (LFPSE) system [1] supports a systems-based approach to learning for patient safety. The former encourages staff to use SEIPS as a key tool for analysing work system factors that both support and threaten safety. LFPSE is designed to provide incident-related data in a format that supports the use of tools such as SEIPS.

### Interpretation within the context of the wider literature

Current approaches to incident analysis often focus on what went wrong because What-You-Look-For-Is-What-You-Find [8]. Exploring healthcare resilience and factors promoting safety within incidents provides a more comprehensive picture of healthcare work systems. Identifying how safety was facilitated uses a different lens to interpret incident report data and can highlight where systems and resilient activities are maintaining safety. This balances the negative and stigmatizing effect of a focus on errors, failures, and unsafe acts [14, 17, 23]. Exploring what went well when performing incident analysis can improve staff morale, support learning, and promote a positive organizational safety culture [24–27].

### Implications for policy, practice, and research

Thematic analysis of the narrative content of incident reports is time-consuming. Due to the volume of incidents reported, those resulting in severe harm or death are often prioritized [2]. Machine learning tools to efficiently gather information from the textual content of incidents are being introduced in England with LFPSE [28]. Natural language processing can efficiently analyse vast quantities of such data and shows promise for learning based on newer concepts of healthcare safety, making a broader approach to incident analysis more feasible [14, 29]. Although systematic use of a Safety-II lens may benefit from machine learning or natural language processing, we have shown that it is possible to extract meaningful data manually whenever incident reports are investigated or analysed. Our study provides early evidence that using a Safety-II lens for reviewing incident report data can offer additional insights.

### Strengths and limitations

This study used a systematic approach to identify facilitators of safety and healthcare resilience within incident reports. Analysis was undertaken by medication safety pharmacists whose clinical insight allowed the interpretation of the narrative descriptions. Anticoagulants were chosen as the focus of the study because they are high-risk medications associated with patient harm and their use involves all aspects of the work system. This allowed insight into a wide range of activities undertaken by staff, patients, and caregivers to maintain safety. Although the study took place using data from the incident reporting system in England, this approach is likely to work in different reporting systems.

There remain challenges in adopting this approach to analysis. Incident reports are written using standard forms and often require knowledge of the clinical and organizational context for interpretation. Furthermore, these are not designed to capture information about the work system or healthcare resilience. Information quality in reports is variable [30, 31], and this analysis depended on the reviewers’ knowledge to draw out information on work-system factors and resilience potentials. Our method would benefit from further testing in a larger study of a contrasting subject area, employing additional reviewers.

## Conclusion

Incident reports can provide vital insight into how safety is facilitated and the role of healthcare resilience. This approach allows the identification of a broader range of potential areas to intervene, complementing traditional incident analysis. Information about resilient activities and the work system factors facilitating safety can be used to proactively highlight and explore opportunities for improvement. Natural language processing and machine learning have the potential to develop this approach, given the greater efficiency for incident analysis these tools can provide.

## Supplementary Material

mzae057_Supp

## Data Availability

The data underlying this article cannot be shared publicly due to the information governance requirement of the participating organizations. The data will be shared on reasonable request to the corresponding author.
