# Supplementary material for: Seeking systems-based facilitators of safety and healthcare resilience: a thematic review of incident reports
Source: Int J Qual Health Care. 2024 Jun 25;36(3):mzae057. doi: 10.1093/intqhc/mzae057 (PMC11233260; doi:10.1093/intqhc/mzae057)
Supplement: mzae057_Supp [file mzae057_supp.zip › Supplementary Information.docx]

## Supplementary Information: Incident reporting system search terms

Medication names included: apixaban, heparin, dalteparin, enoxaparin, rivaroxaban, edoxaban, dabigatran, warfarin, tinzaparin and fondaparinux (along with their associated trade names)

Key terms and abbreviations commonly associated with anticoagulation were: “INR”, “APTT”, “VKA”, “NOAC”, “DOAC”, “LMWH”, Vitamin K, Phytomen*, Anticoag*, Anti-coag*, PCC, Prothrombin and Octaplex.

Abbreviations were not written in full where the components were already included as search terms, for example, the term Low Molecular Weight Heparin (abbreviated as LMWH) would be identified by the term heparin.
